# Preservation of *Bacillus firmus* Strain 37 and Optimization of Cyclodextrin Biosynthesis by Cells Immobilized on Loofa Sponge

**DOI:** 10.3390/molecules17089476

**Published:** 2012-08-08

**Authors:** Rúbia Pazzetto, Sabrina Barbosa de Souza Ferreira, Elder James Silva Santos, Cristiane Moriwaki, Teresinha Aparecida Guedes, Graciette Matioli

**Affiliations:** 1 Department of Pharmacy, State University of Maringá (UEM), Av. Colombo, 5790, Maringá-PR 87020-900, Brazil; 2 Department of Statistics, State University of Maringá (UEM), Av. Colombo, 5790, Maringá-PR 87020-900, Brazil

**Keywords:** microorganism preservation, response surface methodology, cyclodextrin, loofa sponge, cells immobilization, *Bacillus firmus*

## Abstract

The preservation of *Bacillus firmus* strain 37 cells by lyophilization was evaluated and response surface methodology (RSM) was used to optimize the β-cyclodextrin (β-CD) production by cells immobilized on loofa sponge. Interactions were studied with the variables temperature, pH and dextrin concentration using a central composite design (CCD). Immobilization time influence on β-CD production was also investigated. *B. firmus* strain 37 cells remained viable after one year of storage, showing that the lyophilization is a suitable method for preservation of the microorganism. From the three-dimensional diagrams and contour plots, the best conditions for β-CD production were determined: temperature 60 °C, pH 8, and 18% dextrin. Considering that the amount of dextrin was high, a new assay was carried out, in which dextrin concentrations of 10, 15, and 18% were tested and the temperature of 60 °C and pH 8 were maintained. The results achieved showed very small differences and therefore, for economic reasons, the use of 10% dextrin is suggested. Increasing the immobilization time of cells immobilized on synthetic sponge the β-CD production decreased and did not change for cells immobilized on loofa sponge. The results of this research are important for microorganism preservation and essential in the optimization of the biosynthesis of CD.

## 1. Introduction

Microbiological tests are carried out in many industries such as the pharmaceutical, food and clinical ones. These microorganisms need to be preserved for application of defined quantities and to obtain the desired product. In all cases the cost of preservation, maintenance and the length of time cultures remain viable determine the chosen preservation technique [1,2,3]. 

The purpose of this preservation is to keep the cultures without morphological, physiological or genetic changes, as well as maintaining their viability and stability [4]. Drying has been the preferred method for long term storage of cultures for decades, though the technique if not free of cell viability losses. There are several drying methods and, despite the worldwide use of these technologies, no generic method for all applications is known [3]. The current industry standard for preserving microorganisms is freeze drying or lyophilization, and the anhydrous product obtained can be stored, tightly sealed under vacuum or under a protective atmosphere for long periods of time with good activity against rehydration [5].

Preserved microorganisms are usually resuspended in appropriate culture medium and used in the biosynthesis of industrial products. Recently many methods of statistical experimental design have been used in the optimization of bioprocesses. Among them, response surface methodology (RSM) is a viable method to identify the effect of individual variables in order to determine optimal conditions for a multivariable system and increase its efficiency [6]. For many authors, statistical optimization is a common practice and has been a good solution for the optimal values determination of parameters such as pH, temperature, fermentation time, inoculum size, substrates concentration, and other components of the fermentation medium of biotechnological processes [6,7,8]. 

The immobilization of microorganisms on loofa sponge for the production of cyclodextrins (CDs) was studied [9], but until the moment, no reports on the use of RSM to optimize the fermentation conditions have been found in the literature.

CDs are cyclic oligosaccharides composed of glucopyranose units linked by α-(1,4)-glycosidic bonds that form a cylindrical structure with industrial applications in various fields. On account of their hydrophobic cavities, which can incorporate hydrophobic compounds, they can change the physical and chemical properties of guest molecules, such as solubility and stability [10,11,12]. They are produced from starch by the action of the enzyme cyclodextrin glycosyltransferase (CGTase) by a cyclization reaction. CGTase also catalyzes glycosylation reactions, which converts water-insoluble and unstable aromatic compounds into the corresponding water-soluble and stable compounds to improve their bioavailability and pharmacological properties [13,14].

The use of CDs has increased significantly, mainly in the food industry. These molecules have been widely used for stabilization of flavours and protection of sensitive compounds from degradation induced by light or oxygen, thereby increasing the storage time; for vitamins and dyes solubilization; for elimination of unpleasant odours or tastes, microbiological contamination and other undesirable compounds and, for cholesterol removal from animal products thus improving their nutritional characteristics [15].

Cell immobilization is the physical confinement of whole cells with the preservation of certain catalytic activities and has been applied in many biochemical processes [16,17]. Among the advantages of immobilized cell systems are the elimination of extraction and enzyme purification steps, repeated and prolonged use of cells, higher yields of enzyme activity, better operational stability and easy separation of cells from the fermentation medium [16,18]. 

Loofa sponge, obtained from the dried fruit of *Luffa cylindrica*, has been used successfully as a matrix for cell immobilization, because it is a biodegradable material, porous, resistant, and low cost. Furthermore, it is readily available and the immobilization method for this matrix is simple [19,20]. The use of low cost matrices allows the immobilization method to be implemented with minimal additional production cost [21]. Another support recently used in the immobilization of microbial cells is synthetic sponge. This sponge has a polyurethane composition that is resistant and hydrophobic. When used as an immobilization support, it acts as an inert matrix and does not interfere with cell properties [22].

The present work was aimed to evaluate the preservation of *Bacillus firmus* strain 37 cells by lyophilization and apply RSM to optimize the fermentation conditions for the production of β-cyclodextrin (β-CD). In this paper, the optimized parameters were pH, temperature, and dextrin concentration. Influence of immobilization time on β-CD production by cells immobilized on loofa sponge and synthetic sponge was also analyzed. 

## 2. Results and Discussion

### 2.1. Immobilization of *Bacillus firmus* Strain 37 Cells on Loofa Sponge

**Figure 1:**
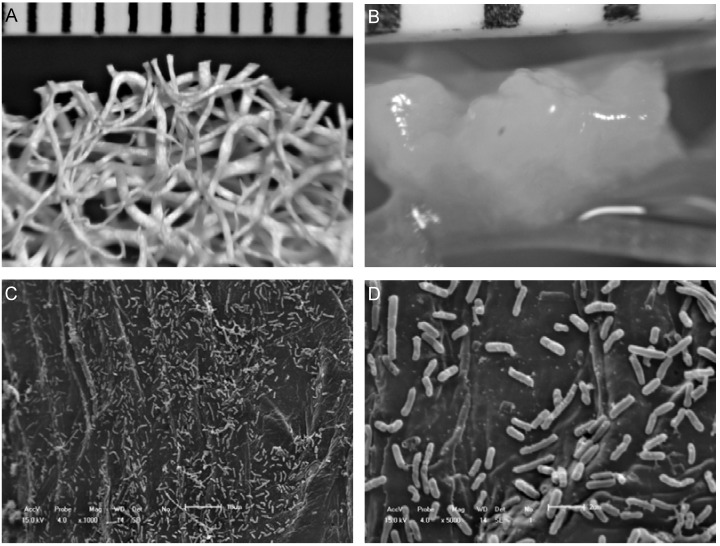
Photographs showing (**A**) uninoculated fibrous network of loofa sponge; (**B**) colony of *B. firmus* strain 37 grown on loofa sponge fibers; and (**C**, **D**) scanning electron micrograph of *B. firmus* strain 37 immobilized on surface of a single loofa sponge fiber. The detail in the superior position of the pictures A and B is a graduated scale in millimeters.

Loofa sponge fibers uninoculated with *B. firmus* strain 37 are shown in Figure 1A. One colony of the bacteria can be observed attached to a fiber with an approximate size of 2 mm (Figure 1B). With the SEM it was possible to verify that a large number of cells adhered to the loofa sponge (Figure 1C,D). The immobilization occurred spontaneously with the adhesion/adsorption microbes on the natural support through electrostatic interactions [23].

The innovative use of loofa sponge, an environmentally friendly matrix, for immobilization of *B. firmus *strain 37 resulted in a high β-CD production, as showed by Pazzetto *et al*. [9]. Since a large number of cells were able to adhere on the surface of the loofa sponge fibers, innovative studies of microorganism preservation and optimization of the β-CD production process are necessary and were conducted in this work.

### 2.2. *Bacillus firmus* Strain 37 Preservation by Lyophilization

**Figure 2:**
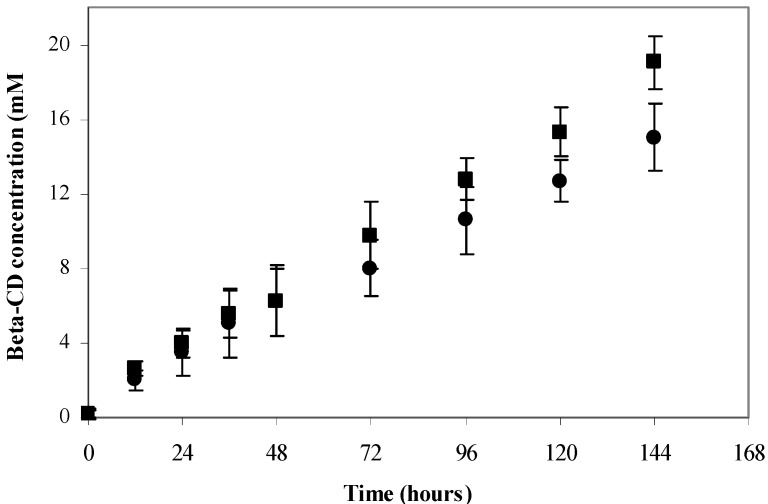
Evaluation of *B. firmus* strain 37 preservation by lyophilization. β-CD production by cells newly lyophilized (■) and after one year of storage (●).

The β-CD production by *B. firmus* strain 37 was similar after one year of storage, reaching a value of 19.04 ± 1.4 mM β-CD after the lyophilization procedure and 15.02 ± 1.8 mM β-CD after one year of storage (Figure 2).

Moriwaki *et al*. [4] used other methods for storing *B. firmus* strain 37: periodical sampling in solid culture medium, addition of a cell suspension in sterile soil and freezing at −70 °C. The methodology of periodical sampling in solid culture medium was not suitable, contrary to the other two methods, which were satisfactory for the storage and preservation of the strain.

Starting from lyophilized cells of *B. firmus* strain 37 and subsequent immobilization on loofa sponge [9], the β-CD production almost doubled compared by the same bacteria frozen and stored at −80 °C and immobilized on silica-titanium (SiO_2_/TiO_2_) and silica-manganese (SiO_2_/MnO_2_) [24], demonstrating the superiority both the immobilization on loofa sponge and the microorganism maintenance method [9].

### 2.3. Optimization of β-Cyclodextrin Production by Immobilized Cells on Loofa Sponge

A statistical experimental design (RSM) was used to optimize the conditions for β-CD production by cells of *B. firmus* strain 37 immobilized on loofa sponge. With the data from this design, a predictive equation was obtained after the analysis of variance (ANOVA), which is an appropriate model for β-CD production and is given below [Equation (1)]:


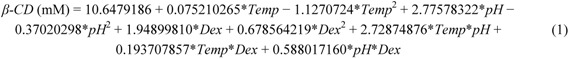
(1)

where *Temp* is the temperature and *Dex* is the dextrin concentration. The coefficient of determination (R^2^) for β-CD production was 0.79, indicating that the statistical model explained 79% of the variability in the response.

**Figure 3:**
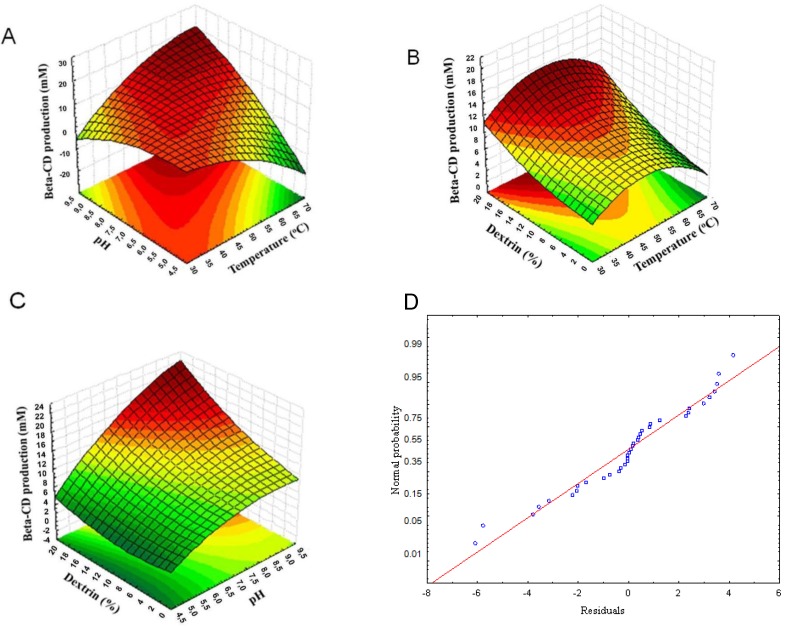
Statistical optimization of β-CD production using RSM. (**A**) pH and temperature; (**B**) dextrin and temperature; (**C**) dextrin and pH; and (**D**) normal probability plot of residuals.

The optimal level of each variable and the effect of their interactions on β-CD production were studied by plotting the three-dimensional diagrams and contour plots of calculated response surfaces from the interactions among the three variables (Figure 3). From these response graphs, it was apparent that the best conditions for this process corresponded to a temperature of 50 °C or above (Figure 3A,B), higher pH values (Figure 3A,C), and the highest dextrin concentrations (Figure 3B,C). When a normal probability plot of residuals was constructed, a selection was made for the normality assumption, which was satisfactory, since nearly all the residuals fell approximately along a straight line (Figure 3D). Considering that the closer the value of R^2^ is to 1.0, the better the model predicts the response, the R^2^ obtained here also shows that the model has acceptable predictive ability.

Vignoli *et al*. [25] constructed a statistical model that could explain 81% of the variability in the response, similar to that obtained in this study (79%); and, bearing in mind that this is a biological experiment, also concluded that the model showed a reasonable predictive ability.

Therefore, from the analysis of Figure 3 and the responses obtained in practice, the optimal process conditions were determined to be temperature 60 °C, pH 8, and 18% dextrin. Considering that the amount of dextrin was high, a new assay was carried out, in which dextrin concentrations of 10, 15, and 18% were tested and the temperature of 60 °C and pH 8 were maintained. The results obtained were 19.26 mM, 19.14 mM, and 20.35 mM of β-CD produced for the dextrin concentrations of 10, 15, and 18%, respectively, which showed very small differences in the β-CD production, so for economic reasons, the use of 10% dextrin is suggested. Thus, the obtained results confirmed that the fermentation process conditions used previously by Pazzetto *et al*. [9] are suitable. In this same work, they evaluated the operational stability of *B. firmus* strain 37 cells immobilized on loofa sponge discs and was reported that at the end of 10 days of process 63% of the initial production of β-CD was maintained. The RSM indicated accuracy and applicability when it was used to optimize the production of α-amylase by *Streptomyces erumpens* cells immobilized on loofa sponge [8]. Kumar and Satyanarayana [26] immobilized sporangiospores of *Thermomucor indicae-seudaticae* on alginate to produce glucoamylase, and used the RSM to optimize the production. The use of optimized parameters led to an increase of 41 to 60% in enzyme production, depending on the type of reactor. In both studies, the statistical models explained around 97% of the variability in the response.

### 2.4. Effect of Immobilization Time of *Bacillus firmus* Strain 37 Cells on Loofa Sponge for β-Cyclodextrin Production

The immobilization time of *B. firmus* strain 37 for a period of one to four days on loofa sponge was evaluated by Pazzetto *et al*. [9], under the same conditions of this study, except that the initial biomass was 70 mg of lyophilized cells. One day of immobilization was enough for the adhesion of bacterial cells to the fibers of the sponge, because there was no difference in the β-CD production between the different times. In this study, prolonged periods of immobilization were evaluated (10, 20 and 30 days) and the β-CD production by cells immobilized on loofa and synthetic sponges was compared. Again, the increase in the immobilization time for cells immobilized on loofa sponge was not effective, showing that the β-CD production is not influenced by the immobilization time.

**Figure 4:**
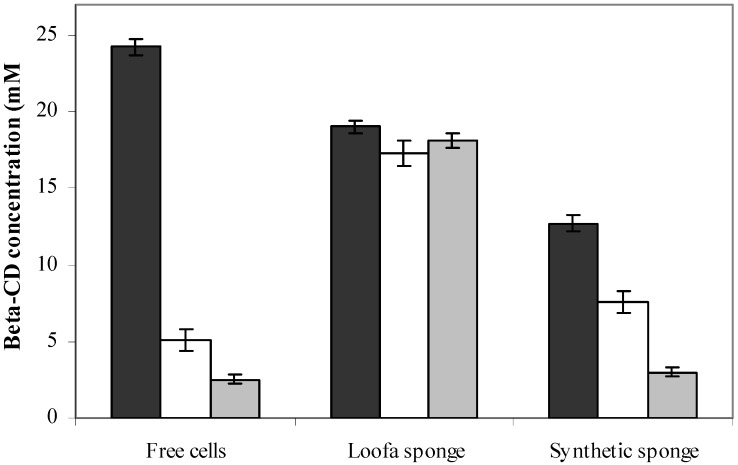
Effect of immobilization time on β-CD production by free and immobilized cells. (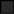) 10 days; (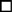) 20 days; and (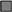) 30 days.

The increase in the immobilization time for cells immobilized on synthetic sponge decreased the β-CD production. A similar behavior was observed with the free cells. However, for cells immobilized on loofa sponge, this increase in the contact time between cells and matrices was indifferent, reaching an average production of 18.14 mM of β-CD (Figure 4).

Similar results were found with the *Streptomyces clavuligerus* and *Fusarium moniliforme* immobilization on loofa sponge. There was no increase in weight of the discs after 48 h of immobilization, suggesting that the spaces of the sponge for microorganisms adhesion were saturated at this time [20,27]. To *Chlorella sorokiniana* uptake of nickel (II) from aqueous solutions, the cells were immobilized on loofa sponge for 10 days and, after, were taken from the culture flasks, washed thoroughly with fresh culture medium to remove any free algal cells, transferred to fresh medium and incubated 24 days for uniform algal growth along the surface of the fibers [28]. 

## 3. Experimental

### 3.1. General

β-Cyclodextrin and phenolphthalein were purchased from Sigma (St. Louis, MO, USA). Soluble starch from potato (article 101,252) was purchased from Merck (Darmstadt, Germany). Maltodextrin (Dextrin 10 from maize starch, article 31,410) was obtained from Fluka (Buchs, Switzerland). Loofa sponges were purchased in local market (Maringá, PR, Brazil). All other chemicals used were of analytical grade. The bacterial suspensions were freeze-dried in a Christ Beta 1–16 freeze dryer. Scanning electron micrographs were obtained from a scanning electron microscope Shimadzu model SS 550 with an acceleration voltage of 10 kV. The β-CD concentrations were measured using a Tecnal SP 1105 spectrophotometer (Piracicaba, Brazil). 

### 3.2. Culture Conditions and Microorganism Reactivation Procedure

A solid medium composed of (% w/v): 1.0 soluble starch; 0.5 polypeptone; 0.5 yeast extract; 0.1 K_2_HPO_4_; 0.02 MgSO_4_·7H_2_O; 0.01 Congo red stain; 1.0 Na_2_CO_3_; and 1.5 agar, was used for cultivation of *B. firmus* strain 37, isolated from the soil of a cassava plantation by Matioli *et al*. [29]. With the colonies formed was prepared bacterial suspensions which were frozen and freeze-dried. The lyophilized cells were stored in a freezer and used in the immobilization procedure. The microorganism reactivation was carried out in 250 mL Erlenmeyer flasks containing 30 mg of lyophilized cells and 50 mL of liquid culture medium of similar composition to that of the solid medium, except for the agar and stain. The flasks were incubated at 37 °C in an orbital shaker (120 rpm) for 24 h.

### 3.3. Immobilization Procedure

The immobilization of *B. firmus* on loofa sponge was carried out according to the method developed by Meleigy and Khalaf [20] for production of gibberellic acid by *F. moniliforme*, with some modifications. Loofa sponge, obtained from the dried fruit of *Luffa operculata* and free of seeds, was cut into discs about 23–25 mm in diameter and 2–4 mm thick. The discs were soaked in boiling water for 30 min, washed thoroughly with tap water, and left for 24 h in distilled water, changed three times. They were then oven-dried at 70 °C and sterilized in an autoclave at 121 °C for 20 min.

For the immobilization of *B. firmus* on synthetic sponge, discs of this matrix were cut in the same size as the loofa discs, put to boiling for 10 min, washed with distilled water, dried at room temperature and sterilized by autoclaving at 121 °C for 20 min [9].

The immobilization process occurred by the addition of three loofa or synthetic sponge discs to the medium containing the reactivated cells. The matrices and bacterial cells stayed in contact for 24 h at 37 °C in an orbital shaker at 100 rpm. To obtain the free biomass, the reactivated cells were separated from the medium by centrifugation and used as controls.

### 3.4. Scanning Electron Microscopy

The cell immobilization on loofa sponge was evaluated by Scanning Electron Microscopy (SEM) using Shimadzu SS 550 microscope (Shimadzu Corporation, Kyoto, Japan) according to the method described by Moriwaki *et al*. [30], using a scanning electron microscope with an acceleration voltage of 10 kV. The pictures were taken of a single fiber after washing.

### 3.5. Evaluation of *Bacillus firmus* Strain 37 Preservation by Lyophilization

*B. firmus* strain 37 was initially grown on solid medium and incubated at 37 °C for 48 h. The colonies formed were removed from the culture medium and a bacterial suspension was prepared. One mL of this suspension was transferred to small glass vials. The samples were frozen and freeze-dried. Then, the lyophilized cells were stored in a freezer. The cells were reactivated, centrifuged and a β-CD production was conducted at 60 °C in an incubator with shaking at 100 rpm, and 10% maltodextrin (w/v) in 50 mM Tris-HCl and 5 mM CaCl_2_, pH 8.0 for 144 h, with 30 mg of cells newly lyophilized and cells with one year of storage. Aliquots of 1 mL were collected daily, diluted in 1 mL of distilled water and boiled for subsequent quantification of the β-CD produced.

### 3.6. Cyclodextrin Production by Immobilized Cells

After the immobilization procedure described above, the matrices were washed in sterile saline and transferred to 250 mL Erlenmeyer flasks containing reaction medium, which consisted of 50 mL of maltodextrin solutions at different concentrations and in combination with different pHs and temperatures, which will be detailed in the following section. The production was conducted for 5 days, in an orbital shaker at 100 rpm. Aliquots of 1 mL were collected after 120 h of assay, diluted in 1 mL of distilled water, and boiled for later quantification of β-CD.

### 3.7. Optimization of Conditions for β-Cyclodextrin Production by Immobilized Cells

The conditions for β-CD production by CGTase from *B. firmus* strain 37 immobilized by adsorption on loofa sponge were optimized by RSM, by selecting the levels of three independent variables: temperature, pH, and dextrin concentration. A factorial central composite design (CCD) was constructed in which each factor was studied at two different levels. For temperature, the levels 40 °C and 60 °C were defined, for pH the levels 6 and 8, and for the dextrin concentration the levels 5% and 15%. These levels were coded as +1 and −1 as shown in Table 1. Two axial points (−α, +α) were added, which corresponded to the temperature levels of 33 °C and 67 °C, the pH levels of 5 and 9, and the dextrin concentration levels of 2% and 18%. In addition to these, the central point was implemented, which corresponded to the levels of 50 °C, pH 7, and 10% dextrin concentration. Fifty mL of CD production medium were prepared in individual 250 mL Erlenmeyer flasks, in different combinations of levels and variables. The experiments were conducted for 5 days as described in the previous section, and the β-CD concentration was determined and taken as the response variable.

**Table 1:** Experimental design and results of CCD for the response surface methodology.

Experiments	Temperature	pH	Dextrin	β-CD Production (mM)	
				Experimental	Predicted
1	−1	−1	−1	8.830	8.540
2	1	−1	−1	6.462	2.846
3	−1	1	−1	7.410	7.458
4	1	1	−1	14.561	12.678
5	−1	−1	1	11.869	10.874
6	1	−1	1	8.879	5.955
7	−1	1	1	11.404	12.144
8	1	1	1	20.727	18.140
9	−α	0	0	9.129	8.288
10	+α	0	0	1.906	8.501
11	0	−α	0	1.640	5.983
12	0	+α	0	12.421	13.833
13	0	0	−α	6.380	9.249
14	0	0	+α	11.875	14.761
15	0	0	0	11.418	10.648
16	0	0	0	11.836	10.648
17	0	0	0	11.889	10.648
18	0	0	0	11.370	10.648
19	0	0	0	12.273	10.648
20	0	0	0	12.202	10.648
21	0	0	0	12.580	10.648
22	0	0	0	11.687	10.648

### 3.8. Influence of Immobilization Time on β-Cyclodextrin Production

β-CD production by cells immobilized on loofa sponge and synthetic sponge was evaluated at different immobilization times. After the reactivation of 30 mg lyophilized cells, three matrices discs were added to the culture media which were kept at 37 °C for 5 days. Then, the discs were removed and transferred to fresh media, where they remained for over 5 days. This process was performed several times until reaching final immobilization times of 10, 20, and 30 days. After immobilization, β-CD production was carried out according to section 3.6, keeping 10% dextrin concentration, temperature of 60 °C, and pH 8. Then, the amount of β-CD produced was determined as described below. The same procedure was performed with free cells.

### 3.9. Colorimetric Determination of β-Cyclodextrin

The assay was carried out by mixing sample (0.5 mL) and 0.06 mM phenolphthalein solution (2.5 mL) containing 0.12 M carbonate-bicarbonate buffer that was prepared at the dosage moment from a stock solution of 3 mM phenolphthalein in 95% ethanol. The absorbance was read at 550 nm and for the blank, the sample was replaced by distilled water. The β-CD concentration was determined by discoloration of phenolphthalein solution which occurs after the complexation with β-CD [30].

### 3.10. Statistical Analysis

The results of β-CD production in the CCD experiments were submitted to analysis of variance and a surface response second order was fit, using the program Statistica 8.0/2008 (Stat Soft, Inc., Tulsa, OK, USA).

## 4. Conclusions

Lyophilization showed to be a suitable method for the microorganism preservation, especially for *B. firmus* strain 37 cells that remained viable for one year of storage. The use of RSM allowed the construction of a statistical model of acceptable predictive ability for β-CD production using immobilized *B. firmus* strain 37 cells on loofa sponge and showed the factorial CCD enabled to find the appropriate parameters for the fermentation process conditions. An increase of the immobilization time of cells on loofa sponge did not change the β-CD production. The results obtained in this research are relevant because microorganism preservation from different sources is important for many areas of research and clinical sciences, such as microbiology and biotechnology. Besides, the optimization of the fermentation process is essential in these areas and for many industries. 
